# Review of: Ailing in Place: Environmental Inequities and Health Disparities in Appalachia

**DOI:** 10.13023/jah.0301.06

**Published:** 2021-01-24

**Authors:** Jerome A. Paulson

**Keywords:** Appalachia, environmental health, health disparities, book review

## Abstract

The *Journal of Appalachian Health* is committed to reviewing published media that relates to contemporary concepts affecting the health of Appalachia. The Appalachian environmental inequities and the health disparities we face have a direct effect on our experience of illness. Dr. Jerome Paulson reviews the book *Ailing in Place: Environmental Inequities and Health Disparities in Appalachia*.

## MEDIA TYPE: BOOK

### CITATION

Morrone M. *Ailing in Place: Environmental Inequities & Health Disparities in Appalachia*. Ohio University Press, 2020.

ISBN-13: 978-0-8214-2420-9; Cost: $ 55.00 Hardcover, $26.95 Kindle edition, $26.95 Paperback

### ABOUT THE REVIEWER

Jerome A. Paulson, MD, FAAP (he, him and his) Professor Emeritus of Pediatrics and of Environmental & Occupational Health, George Washington University School of Medicine and Health Sciences and George Washington University Milken Institute School of Public Health. Dr. Paulson has spent his career teaching and practicing primary care pediatrics at Case Western Reserve University and George Washington. He taught about children’s environmental heath at the GW Milken Institute School of Public Health. He currently consults about the health impact of climate change and advocates for climate solutions that are health solutions.

### ABOUT THE AUTHOR

Dr. Michele Morrone holds a PhD in environmental planning from The Ohio State University, an MS in forest resources from the University of New Hampshire and a BS in natural resources from The Ohio State University. At Ohio University, Dr. Morrone is an Associate Professor of Environmental Health, the Director of Environmental Studies, and the Associate Director of Academic Affairs at the Voinovich School. Dr. Morrone previously served as the Chief of the Office of Environmental Education at Ohio EPA. Morrone, has spent 25 years working in Appalachia and studying and teaching about the area. For this book she interviewed hundreds of health professionals throughout the area.

### THE REVIEW

Using the personal stories of individuals living in Appalachia and of public health professional working there, Dr. Morrone paints the very bleak picture of the influence of environmental factors on health. So much has been made of the responsibility of individuals for our own health including diet, exercise, and tobacco use. While all of these factors are under individual control and do influence health, so many other systemic community and environmental factors impact health. For example, whether there is a community water or septic system, what polluting industries are invited into an area, and the availability of resources including healthcare services and basic infrastructure also influence health. These problems and more are so prevalent in the huge swathe of territory stretching from western New York State to northeastern Mississippi and negatively influence the health of those who are and have lived there.

This book should be read by anyone practicing and studying health-related professions and those wanting to influence public policy. Health professionals in Appalachia will have lived some of the stories in this book. However, learning about the experiences of individuals living in one of the other 420 counties in 13 states is likely to inform the work being done locally. To understand all of the efforts that have gone into trying to help the citizens of Appalachia that have fallen short can also inform future efforts. For those wanting to inform and change local, state, and national policy to improve the situation in Appalachia, this book contains both good stories and important facts. Use of good stories is so important to policy making. These personal memorable examples motivate people and legislators to act. The facts, which are also presented in this book, become important behind the scenes when the policies are developed and implemented.

Dr. Morrone reviews the influence of the built environment: how water, food, pollution, resource extraction, and disasters impact the health of those who live in Appalachia. These presented lessons certainly extrapolate to other under-resourced areas with very sparce populations such as Native American Reservations or rural areas of the mid-west.

The way Dr. Morrone melds stories and facts is a unique approach to scholarship that makes this book stand out from others on a similar topic. Chapter Two begins with the following sentence: “Forest County in Western Pennsylvania is a compelling case study of the built environment in Appalachia.” The author tells the story of building a prison in Forest County as a means of providing good paying jobs in an area with very little in the way of economic opportunity. Layered on that story is a scholarly discussion of the key concept of the built environment. The author points out that much of that theory, developed around urban and suburban settings, doesn’t work in rural Appalachia. Implementation of those precepts leads to development which does not provide the economic or social benefits desired and which does not benefit the people who live in Forest County.

In Chapter Three, “A Place for Food,” the author describes the situation of Athens County, Ohio, the poorest county in Ohio. The inequal access to food in the agricultural setting is enormous. For those who are well to do and who have personal means of transportation, healthy food in appropriate quantiles is available. For most of the residents of the county who are poor and who have limited to no means of transportation, the food deserts are vast. The various federal food programs such as SNAP are ineffective in this type of rural area because there is no accessible place to buy healthy food. The public health departments which are tasked with insuring food safety are understaffed and under-resourced. This theme is repeated over and over in the book. The states and counties through out Appalachia do not have the resources to deal with the problems presented. In addition, the people of Appalachia are so spread out that bringing them services presents overwhelming challenges. These are the stories and the facts that Morrone tells so well in this book.

The book includes extensive endnotes. There are appendices describing the types of public health professionals in each of the 13 states and which state agencies have responsibility for environmental health issues in the state. In addition, for faculty using the book to teach a course, there are an excellent set of discussion questions for each chapter.

This reviewer had some specific complaints directed to the Ohio University Press. The font used in this book is very small. There is not a lot of contrast between the inks used and the buff-colored paper. These issues taken together can make reading the book physically difficult for people “of a certain age.”

